# MindLink-Eumpy: An Open-Source Python Toolbox for Multimodal Emotion Recognition

**DOI:** 10.3389/fnhum.2021.621493

**Published:** 2021-02-19

**Authors:** Ruixin Li, Yan Liang, Xiaojian Liu, Bingbing Wang, Wenxin Huang, Zhaoxin Cai, Yaoguang Ye, Lina Qiu, Jiahui Pan

**Affiliations:** ^1^School of Software, South China Normal University, Guangzhou, China; ^2^Pazhou Lab, Guangzhou, China

**Keywords:** multimodal emotion recognition, multitask convolutional neural network (CNN), support vector machine (SVM), subject-independent method, long short-term memory network (LSTM)

## Abstract

Emotion recognition plays an important role in intelligent human–computer interaction, but the related research still faces the problems of low accuracy and subject dependence. In this paper, an open-source software toolbox called MindLink-Eumpy is developed to recognize emotions by integrating electroencephalogram (EEG) and facial expression information. MindLink-Eumpy first applies a series of tools to automatically obtain physiological data from subjects and then analyzes the obtained facial expression data and EEG data, respectively, and finally fuses the two different signals at a decision level. In the detection of facial expressions, the algorithm used by MindLink-Eumpy is a multitask convolutional neural network (CNN) based on transfer learning technique. In the detection of EEG, MindLink-Eumpy provides two algorithms, including a subject-dependent model based on support vector machine (SVM) and a subject-independent model based on long short-term memory network (LSTM). In the decision-level fusion, weight enumerator and AdaBoost technique are applied to combine the predictions of SVM and CNN. We conducted two offline experiments on the Database for Emotion Analysis Using Physiological Signals (DEAP) dataset and the Multimodal Database for Affect Recognition and Implicit Tagging (MAHNOB-HCI) dataset, respectively, and conducted an online experiment on 15 healthy subjects. The results show that multimodal methods outperform single-modal methods in both offline and online experiments. In the subject-dependent condition, the multimodal method achieved an accuracy of 71.00% in the valence dimension and an accuracy of 72.14% in the arousal dimension. In the subject-independent condition, the LSTM-based method achieved an accuracy of 78.56% in the valence dimension and an accuracy of 77.22% in the arousal dimension. The feasibility and efficiency of MindLink-Eumpy for emotion recognition is thus demonstrated.

## Introduction

Emotions are biological states associated with the nervous system (Damasio, 1998), and its changes are related to subjective feelings and objective behavioral responses. Emotion recognition plays an essential role in human–computer interaction. It is an emerging interdisciplinary research field that covers various methods and techniques in artificial intelligence (AI), natural language processing (NLP), and cognition and social sciences (Poria et al., 2017). Although the studies on emotion recognition have made great improvements in recent years, there are still limitations such as low accuracy and subject dependence. Thus, there is an urgent need for an innovative toolbox with effective methods to enlarge the dataset and improve the accuracy of emotion recognition.

Previous studies (Davidson et al., 1990) exerted the Approach/Withdrawal index as an emotional indicator of the relationship between emotion, approach, and withdrawal. Gianluca et al. (Di Flumeri et al., 2017) demonstrated the reliability of this index of pleasantness. Afterward, some scholars found that algorithms based on transfer learning, fusion of multimodal information, or subject-independent methods can improve the performance of emotion recognition. For example, Nguyen et al. (2018) proposed a novel transfer learning approach based on PathNet and conduct various experiments on the Surrey Audio-Visual Expressed Emotion (SAVEE) dataset and the eNTERFACE dataset and found that this approach could improve the performance of emotion recognition. Sebe et al. (2005) conducted a survey and pointed out that multimodal emotion recognition (such as the combination of facial information, voice, and physiological signals) achieved higher accuracy than traditional single-modal emotion recognition. Furthermore, Georgieva et al. (2015) compared six unsupervised machine learning methods and performed experiments for intersubject models and intrasubject models. The results showed that event-related potential (ERP) clustering (especially the Fuzzy C-means clustering) algorithm was a promising approach that can extract statistical underlying correlations of brain activity among subjects to decode the human emotional state. However, all the above studies did not combine the electroencephalogram (EEG) modality using deep learning technology in subject-independent emotion recognition.

Furthermore, the influence of emotion is manifested in a variety of levels and modalities. On the one hand, peripheral signals (such as facial expressions, verbal speech, and body language) are related to the somatic nervous system and can reflect changes in emotion states. On the other hand, many studies often assessed the power spectra of EEG in different frequency bands to examine their relationship with emotional states. For example, literature (Alsolamy and Fattouh, 2016; Guzel Aydin et al., 2016; Jiang et al., 2019) reported several spectral changes and brain regions related to emotional responses, such as the changes of theta (θ: 4–7 Hz) power in the right parietal lobe, the asymmetry of alpha (α: 8–13 Hz) power in the anterior area of the brain, the asymmetry of beta (β: 14–30 Hz) power in the parietal region, and the changes of gamma (γ: 31–50 Hz) power in the right parietal regions. Most of the previous studies have used peripheral signals or brain signals alone to identify emotions, and little attention has been paid to the fusion between the brain and peripheral signals.

To overcome the abovementioned difficulties in emotion recognition, we proposed an open-source free toolbox named MindLink-Eumpy. MindLink-Eumpy mainly focuses on the recognition of continuous movie-induced emotions rather than discrete emotions (Yan den Broek, 2013) (the basic discrete emotions include happiness, sadness, surprise, fear, anger, and disgust) and provides a series of tools for physiological data processing. MindLink-Eumpy applies vector models to classify emotions because they can quantify emotions better than circumplex models (a concept of discrete emotions) (Posner et al., 2005). Furthermore, MindLink-Eumpy adopts a valence–arousal model (Lang et al., 1997) (continuous emotion) and self-assessment manikins (SAMs) (Bradley and Lang, 1994) to evaluate emotion. The scores of valence and arousal dimensions are both between 1 and 9 (all scores are integers). The valence reflects the level of pleasure, and arousal reflects the level of intensity. High scores represent high levels of pleasure or intensity.

MindLink-Eumpy provides a series of continuous emotion recognition methods based on facial expressions and EEG signals. Specifically, as a toolbox designed for scientific research, MindLink-Eumpy is suitable not only for emotion recognition based on the public databases Database for Emotion Analysis Using Physiological Signals (DEAP) (Koelstra et al., 2012) and MAHNOB-HCI (Soleymani et al., 2012) but also for emotion recognition based on self-created databases. To acquire physiological data and create our own database, MindLink-Eumpy provides recorders (programs for device control, especially programs for collecting data) to collect EEG signals and facial images. Moreover, MindLink-Eumpy implements an event-related potential (ERP) (Sur and Sinha, 2009) paradigm in a Web-based framework to induce the subject’s emotions through video clips. In the detection of facial expression, MindLink-Eumpy uses multitask convolutional neural networks (CNNs) (Lawrence et al., 1997) based on transfer learning techniques to overcome the common problem of lack of data and achieve higher accuracy. MindLink-Eumpy offers two methods in the detection of EEG. One is a subject-dependent model based on support vector machine (SVM) (Cortes and Vapnik, 1995), which is able to achieve high accuracy when the validation data and the training data are homogeneous. The other one is a subject-independent model based on long short-term memory network (LSTM) (Hochreiter and Schmidhuber, 1997; Koelstra et al., 2012), which is used to reduce the effects caused by the individual variations and non-stationarity of EEG signals. The latter method yields more stable performance when the validation data and training data are heterogeneous. Moreover, to improve the accuracy of emotion recognition for homogeneous data, MindLink-Eumpy proposes two decision-level fusion methods for multimodal emotion recognition tasks, namely, weight enumerator and adaptive boosting (AdaBoost) technique (Das et al., 2015), to fuse the decision-level information of SVM and CNN. For the heterogeneous data, the subject-independent method we used is the EEG-based LSTM model. Our experimental results show that when the validation data and training data are homogeneous, the highest average accuracy achieved by the multimodal subject-dependent models in the arousal and valence dimensions were 72.14% and 71.00%, respectively. However, when the validation data and training data are heterogeneous, the highest average accuracy achieved by the EEG-based subject-independent model in the arousal and valence dimensions were 77.22% and 78.56%, respectively.

This paper introduces an open-source Python toolbox for multimodal emotion recognition, MindLink-Eumpy, including its structure, related algorithms, and functions. The *Introduction* section of this paper covers the background and significance of this work. The *Related Work* section introduces the related works on emotion recognition and some related toolboxes. The *MindLink-Eumpy: Architecture, Modules*, *and Features* section presents MindLink-Eumpy and describes its structure, methods, and functions in detail. The *Methods for Emotion Recognition* section describes the continuous emotion recognition methods used in MindLink-Eumpy in detail and proposes multimodal subject-dependent methods and EEG-based subject-independent methods. The *Experiments and Results* section demonstrates the innovations and effectiveness of MindLink-Eumpy. The *Discussion and Conclusion* section summarizes the advantages and limitations of MindLink-Eumpy compared with other state-of-the-art methods, as well as its potential applications and areas of future work.

## Related Work

In this section, we briefly review some related work on emotion recognition. This section includes three subsections: **(i)** related studies on emotion recognition, **(ii)** software-related emotion recognition, and **(iii)** comparison of related software with MindLink-Eumpy.

### Related Research on Emotion Recognition

It is well known that emotion recognition techniques have yielded considerable improvements in the past few years, and here come some articles that inspired us during our study. To begin with, transfer learning technique has the potential to tackle the difficulties of small datasets in emotion recognition area. Facial expression recognition needs plenty of facial images but it is hard to recruit enough subjects. To deal with this problem, Prakash et al. (2019) proposed an automatic facial emotion recognition method using CNNs with a transfer learning approach. This approach was demonstrated to be effective with an average accuracy over 98% in their experiments. Furthermore, we tried to combine facial expression and EEG because the performance of multimodal emotion recognition methods is superior to that of single-modal methods, which has been demonstrated long before. As an example, in a research in 2008: Kessous et al. (Castellano et al., 2008) integrated information from facial expressions, body movements, gestures, and speech and found that the multimodal approach improved accuracy by more than 10% compared to the most successful single-modal system. Finally, we tried to improve subject independence of EEG-based methods because MindLink-Eumpy attaches more importance to human neuroscience. The subject-independent emotion recognition based on EEG signals is the current research hotspot. Under this circumstance, Alhagry et al. (2017) proposed an end-to-end LSTM-recurrent neural network (RNN) to analyze emotion from raw EEG signals, in which they achieved average accuracy rates of 85.65%, 85.45%, and 87.99% in classification for the arousal, valence, and fondness dimensions, respectively. Therefore, we theoretically chose LSTM as the first subject-independent method of MindLink-Eumpy.

### Software Toolboxes Related to Emotion Recognition

This subsection briefly introduces three software toolboxes for emotion recognition that are currently used in both scientific research and industrial applications.

#### Computer Expression Recognition Toolbox (CERT)

The Computer Expression Recognition Toolbox (CERT) (Littlewort et al., 2011) is an open-source free software tool for fully automatic real-time facial expression recognition. It can automatically code the intensity of 19 different facial actions from the Facial Action Unit Coding System (FACS) and six different prototypical facial expressions. Moreover, this tool can estimate the positions of 10 facial features and the 3D orientation (yaw, pitch, and roll) of the head. Previous experiments have demonstrated that CERT can achieve an accuracy of nearly 80% when applied to a spontaneous facial expression dataset (Littlewort et al., 2011).

#### MixedEmotions

The MixedEmotions toolbox (Buitelaar et al., 2018) contains text, audio, and video processing functions aimed at emotion recognition and provides a plug-and-play and ready-to-use set of emotion recognition modules. The current version is mainly applied to three real-world cases: emotion-driven smart TV use (emotion-based recommendation), brand reputation analysis (monitoring the reputation of a brand from tweets and YouTube videos), and call center monitoring (monitoring the emotions of customers in a help desk setting).

#### Toolbox for Emotional Feature Extraction From Physiological Signals (TEAP)

The Toolbox for Emotional Feature Extraction from Physiological Signals (TEAP) (Soleymani et al., 2017) is an open-source MATLAB toolbox that can process and calculate emotion-related features from multiple physiological signals, including EEG, galvanic skin response (GSR), electromyogram (EMG), skin temperature, respiration pattern, and blood volume pulse information. The toolbox has been tested on the MAHNOB-HCI and DEAP databases and has shown promising performance (Soleymani et al., 2017).

### Comparison With MindLink-Eumpy

This subsection compares the differences between software toolboxes for emotion recognition and describes the advantages of MindLink-Eumpy. Table 1 lists the programming languages and functions of the above toolboxes.

**TABLE 1:** Comparison of different toolboxes related with emotion recognition.

Toolbox Name	Programming Language	Main Features
CERT (Littlewort et al., 2011)	Python	- Fully automatic facial expression recognition in real time - Automatically encodes the intensity of 19 different facial actions from FACS and estimates the positions of facial features and the 3D orientation of the head
MixedEmotions (Buitelaar et al., 2018)	Python	- Provides a plug-and-play and ready-to-use set of emotion recognition modules - Provides a unified solution for large-scale emotion analysis on heterogeneous, multimodal, text, speech, video, and social media data streams
TEAP (Soleymani et al., 2017)	MATLAB, Octave	- Imports, processes, and visualizes physiological signals - Processes and calculates emotionally relevant features
MindLink-Eumpy	Python	- Two approaches of facial expression and EEG for emotion recognition - Two decision-level fusion methods for fusion of sub-classifiers in different modalities to improve accuracy - Reads, processes, visualizes multimodal real-time data (facial images and EEG signals) and stores data into a folder system - Subject-independent emotion recognition approach based on LSTM in EEG modality

*CERT, Computer expression recognition toolbox; FACS, Facial action unit coding system.*

MindLink-Eumpy is an open-source Python toolbox with modular tools and frameworks for different functions. The main functions are **(i)** providing a framework for online ERP experiments, **(ii)** reading real-time data from devices during online experiments and practical usage scenario, **(iii)** processing multimodal data including facial images and EEG signals, **(iv)** providing model training interfaces and datasets storage medium (here we call it a database), and **(v)** real-time emotion recognition and data visualization.

By combining decision-level information of facial expressions and EEG, MindLink-Eumpy has obtained a promising accuracy of emotion recognition. Moreover, MindLink-Eumpy provides an LSTM model based on EEG for subject-independent emotion recognition. The abovementioned toolboxes represent the state-of-the-art software in emotion recognition area. While most of the toolboxes provide tools for data processing or methods for emotion recognition, seldom do they focus on the scientific research on human neuroscience and practical applications. MindLink-Eumpy provides tools for EEG collection, preprocessing, and display so as to reflect emotions straightforward. To enhance stability in practical application, MindLink-Eumpy provides tools for facial images, including functions of images processing based on OpenCV and a CNN model for emotion recognition.

## Mindlink-Eumpy: Architecture, Modules, and Features

**FIGURE 1:**
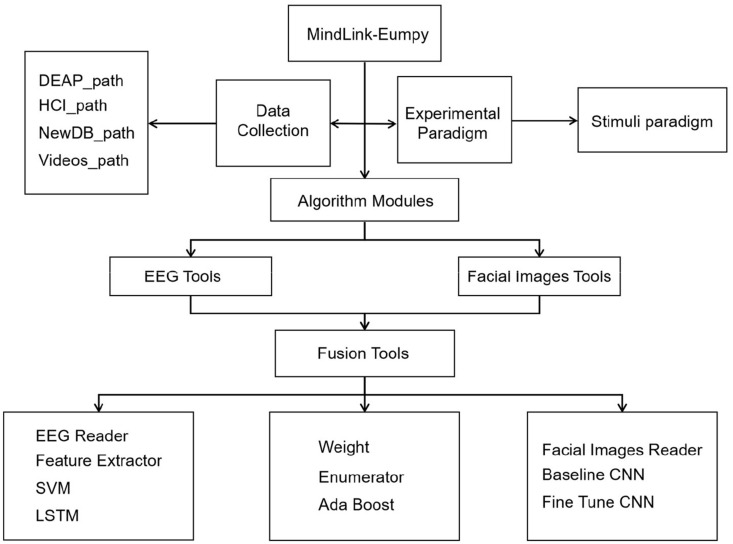
Architecture of MindLink-Eumpy.

This section gives an overview about MindLink-Eumpy, including **(i)** the architecture of MindLink-Eumpy shown in Figure 1, **(ii)** the modules in MindLink-Eumpy, and **(iii)** the features of MindLink-Eumpy. In real-time running, fusion tools are used in the step of fused scores.

### Architecture of MindLink-Eumpy

MindLink-Eumpy can be mainly separated into two parts: database creation (online experiment paradigm) and real-time detection framework. In the modules of database creation, MindLink-Eumpy provides a series of tools for data streaming and decoding from devices (brain–computer interfaces and cameras). During the online experiment, data from the subject will be stored into a folder system (here, we call it a database or a data storage medium). In real-time detection framework, MindLink-Eumpy provides visualization function for EEG, facial images, and analyses of emotion.

Specifically, MindLink-Eumpy utilizes existing Python open-source libraries such as Numpy (van der Walt et al., 2011), scikit-learn, TensorFlow, Keras (Pedregosa et al., 2011), Flask, Pandas, and others. In the database module, we adopted two public emotion databases, i.e., DEAP and MAHNOB-HCI, and created our own database to evaluate the performance of the proposed methods. Emotions are elicited by video clips from commercial films. In the experimental paradigm module, videos in the database are applied to elicit emotion, during which MindLink-Eumpy can use the readers (an EEG reader in the EEG toolbox and a facial image reader in the facial images toolbox) to read and save physiological data. The details of the experiments and evaluation are presented in the *Experiments and Results* section. In the algorithm modules, we integrated all methods into three packages: facial images tools, EEG tools, and fusion tools. In addition to data processing methods, MindLink-Eumpy also provides emotion classification methods, including the multitask CNN method in the facial image toolbox, the SVM and an LSTM in the EEG toolbox, and two methods in the fusion toolbox for decision-level fusion. More details of these methods are shown in *Methods for Emotion Recognition* section.

### Modules of MindLink-Eumpy

#### Database Creation

To address the lack of data problem, we designed a data collection framework to acquire and store facial images and EEG signals. By this module, MindLink-Eumpy can help conduct online experiments and obtain subjects’ data more simply. New methods can be validated more effectively in public databases and our own database. One disadvantage about public databases is that subjects’ emotion and related feedback may differ from culture, gender, and other uncontrollable factors. Therefore, our research lacks data from subjects similar to actual users of MindLink-Eumpy. The function of database creation aims at eliminating the problem of low accuracy of practical application caused by domain differences of data.

##### Device invocations

Physiological data streams are first recorded by hardware such as EEG acquisition equipment (e.g., Emotiv EPOC+ headset in this study) and optical camera. Then, the hardware sends data streams to the back end of MindLink-Eumpy. This kind of process is called data streaming, and we designed programs called readers to conduct the data streaming process. MindLink-Eumpy has two readers (an EEG reader and a facial image reader) to obtain EEG signals and facial images. The EEG reader invokes the driver of the Emotiv EPOC+ headset suitable for MindLink-Eumpy and uses the interfaces of the corresponding software development kit (SDK) to obtain EEG data and store it in the computer memory. The main process of facial image reading is the same as the reading process of EEG signals, but the facial image reader uses the OpenCV library to start the camera and obtain digital images stored in the computer memory.

##### Data storage

MindLink-Eumpy provides a prototype storage medium for data relating to emotions. In the computer memory, MindLink-Eumpy establishes a queue for temporary data storage. By controlling the size of this queue, MindLink-Eumpy is able to synchronize the frequency of data refreshing by devices and the Python-Flask back end to prevent data explosion. However, in external memory, from the perspective of the file format, raw EEG signals are required to be stored in (^∗^.fif) files, but power spectral density (PSD) data (Ng et al., 2019) of the EEG signals are stored as matrices in (^∗^.npy) files through the Numpy library. For facial images, MindLink-Eumpy uses OpenCV to save temporary images as videos in (^∗^.mp4) files. Ground-truth labels and the personal information of subjects are saved in (^∗^.csv) files through the Pandas library. We can access databases with a string of the subject’s information (reported before an experimental trial starts).

#### Real-Time Detection Framework

We designed a real-time detection framework to widen the scope of application of MindLink-Eumpy. Based on data readers, the Python-Flask back end, and web technology, this framework applies the E-Charts technique to visualize real-time data.

##### Electroencephalogram detection

**FIGURE 2:**
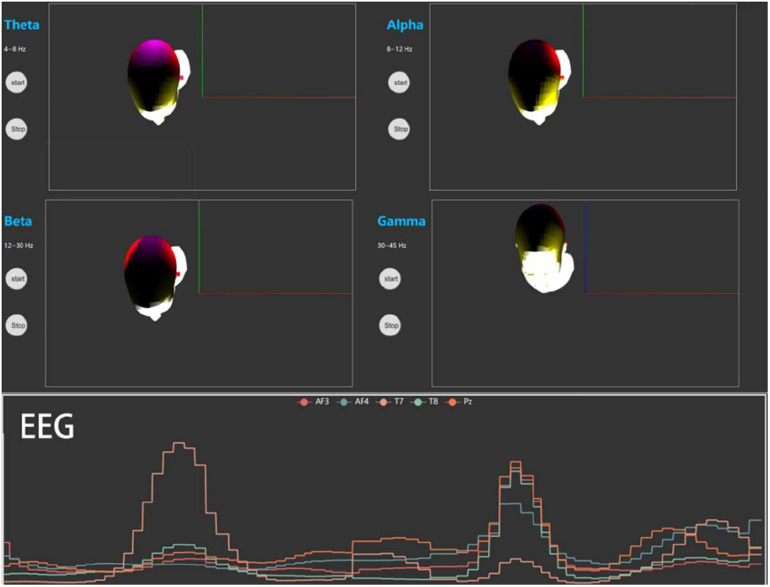
Visualization for power spectral density (PSD) and electroencephalogram (EEG).

In EEG detection, MindLink-Eumpy first modifies the real-time EEG data temporarily stored in the computer memory into a specific format and sends to the front end. Then, the EEG data are displayed on a web page with a visually appealing style. The lower panel of Figure 2 shows the EEG signals of five channels (AF3, AF4, T7, T8, and Pz) in a two-dimensional coordinate system, and the upper panel shows the mapping of PSD data (theta, alpha, beta, and gamma) on brain patterns to reflect the effects of valence and arousal levels in different brain regions. In graphical user interface (GUI), different colors represent different brain regions, and the brightness of the color represents the value of the PSD. Specifically, yellow represents the region where AF3 and AF4 are located, red represents the region where T7 and T8 are located, and purple represents the area where Pz is located. In this study, five channels (AF3, AF4, T7, T8, and Pz) were selected for the default display. Users can also manually select other channels based on their equipment. Furthermore, PSD is the most commonly used feature in emotion recognition (Park et al., 2013). Thus, in the current version of MindLink-Eumpy, we only provide the function of PSD pattern.

##### Facial expression detection

**FIGURE 3:**
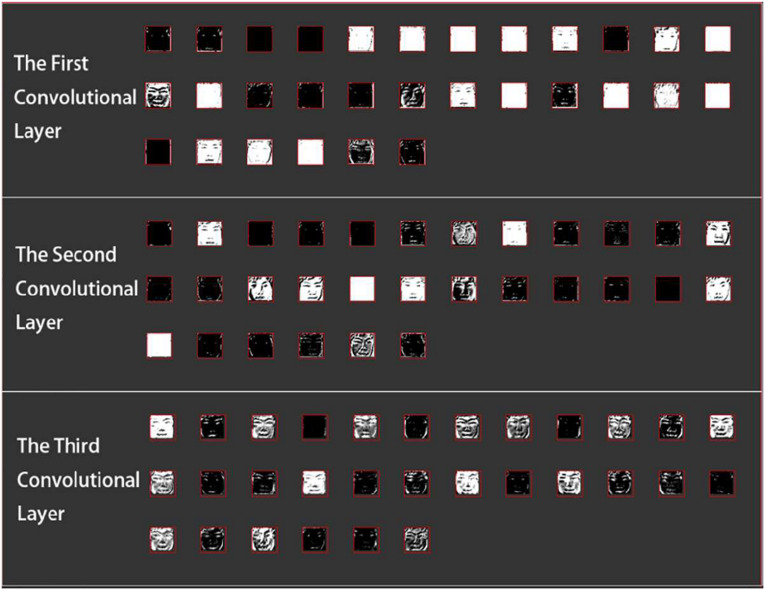
Visualization of facial detection and feature identification by a convolutional neural network (CNN).

In real-time facial image detection, MindLink-Eumpy first uses the Viola–Jones face detector (Viola and Jones, 2001) to detect the face of the subject. Then, facial features are identified by the multitask CNN. Figure 3 shows the calculation results of three layers in the CNN during a forward pass on a web page. For the first and second convolutional layers, the low-level features such as edges and light are displayed. For the final convolutional layer, the high-level features such as the eyes and mouth of a user are displayed.

##### Emotion visualization

**FIGURE 4:**
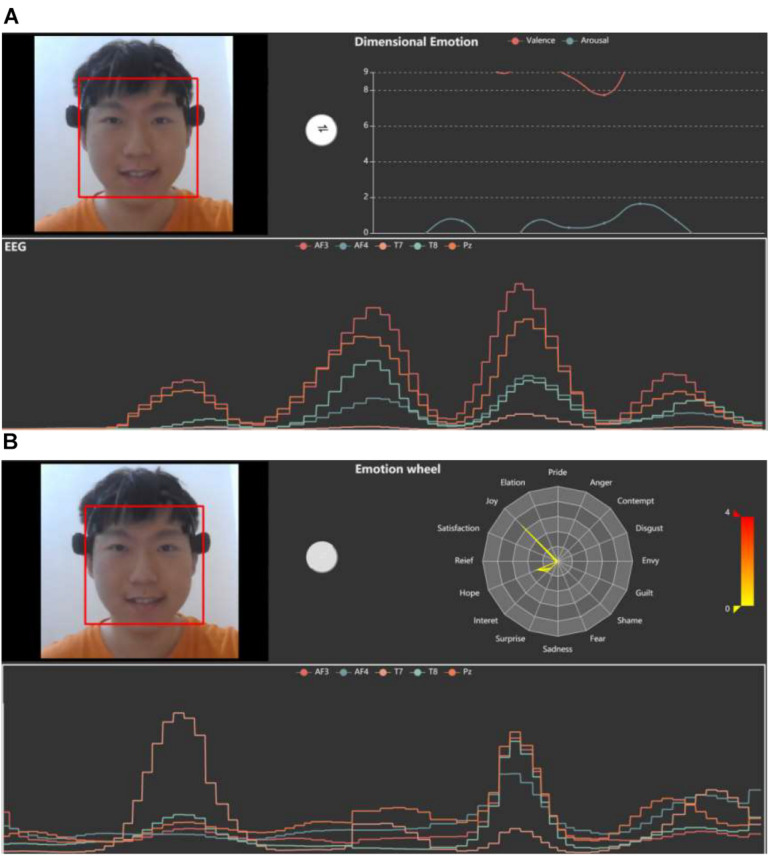
Visualizations for continuous emotion and discrete emotion. **(A)** Graphical user interface (GUI) for continuous emotion. **(B)** GUI for discrete emotion.

Figure 4 shows the main screen of the MindLink-Eumpy’s GUI, which provides visualization functions for EEG data, facial images, continuous emotions (valence–arousal emotion model), and discrete emotions. Continuous emotions can be obtained by fusion methods that combine the predictions of the SVM and CNN in decision level. In this study, the K-nearest neighbors (KNN) was used to transform the continuous emotions to 16 discrete emotions, including pride, elation, joy, satisfaction, relief, hope, interest, surprise, sadness, fear, shame, guilt, envy, disgust, contempt, and anger. Specifically, 16 samples with ground-truth labels in the dataset were first set, and then 16 categories were classified according to Euclidean distance. This function is designed to intuitively display emotions in GUI for users. The intensity of emotions is plotted on a radar map (emotion wheel). In MindLink-Eumpy, both continuous and discrete emotions are sent from the back end to the front end and are displayed in real time. Visual emotion data are displayed in the upper right of the screen. We can click a white button in the middle of the screen to switch interfaces between continuous emotion and discrete emotion.

### Features of MindLink-Eumpy

Herein, we summarize the features of our toolbox. Notably, the toolbox is characterized by simple data acquisition and storage, high accuracy based on multimodal emotion recognition, low algorithmic complexity, and subject independence. We have established a framework for academic experiments, and MindLink-Eumpy provides tools for data streaming, processing, and storage. Moreover, MindLink-Eumpy provides machine learning algorithms and deep learning techniques with promising performance based on both accuracy and algorithm complexity. These accurate methods are based on multimodal emotion recognition, and subject independence is achieved with single-modal EEG data. Furthermore, users can save and access collected data and models that have been trained to customize databases and methods. In short, our toolbox incorporates database, experimental paradigm, and software tools, which allow developers to easily extend model functionality and optimize usability in collaborative development.

## Methods for Emotion Recognition

### Workflows of the Emotion Recognition Methods

**FIGURE 5:**
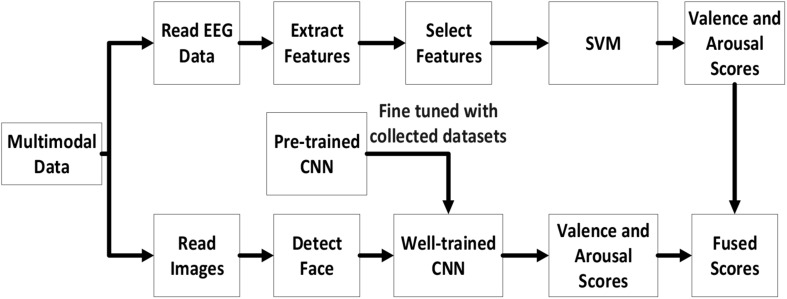
Workflow of the subject-dependent approach in MindLink-Eumpy.

This section describes the multimodal emotion recognition methods of subject dependence, which are provided in the algorithm modules of MindLink-Eumpy, as shown in Figure 5. In the facial image tools submodule, the baseline CNN was pre-trained with a large open-source database, and the CNN modified with a database generated by the authors was a well-trained multitask model. Next, in the EEG tools submodule, the features of data were extracted with a feature extractor and were fed to the SVM and LSTM network. Finally, in the fusion tools submodule, the weight enumerator and AdaBoost method were applied to combine the predictions of the SVM (EEG modality) and CNN (facial expression modality) in decision level. It is worth mentioning that the combination of the SVM and CNN is subject dependent. Therefore, in the experiments, we trained one particular model for each subject separately.

### Facial Expression Detection

#### Architecture of Multitask Convolutional Neural Network

We used a kind of transfer learning technique with a public database and our database to train the multitask CNN model for feature extraction and emotion classification. To obtain a well-trained multitask CNN, first, we pre-trained the CNN using a large regular database FER-2013 with image-level annotations (Goodfellow et al., 2015). Second, we froze all the parameters of the baseline CNN (the first three convolutional layers) and conducted a stochastic gradient descent (SGD) training (fine-tuning) by using a specific small database, while setting the learning rate to 0.0001.

In real-time detection, the images extracted from a video were input into the well-trained multitask CNN, so that we obtained multiple sets of valence and arousal scores. The highest scores were chosen to be the final valence and arousal scores. In addition, we resampled the videos to 4 Hz and used OpenCV to obtain grayscale images (640 × 480 PNG). Then, the Viola–Jones face detector was used to find the facial position in the image frame.

**FIGURE 6:**
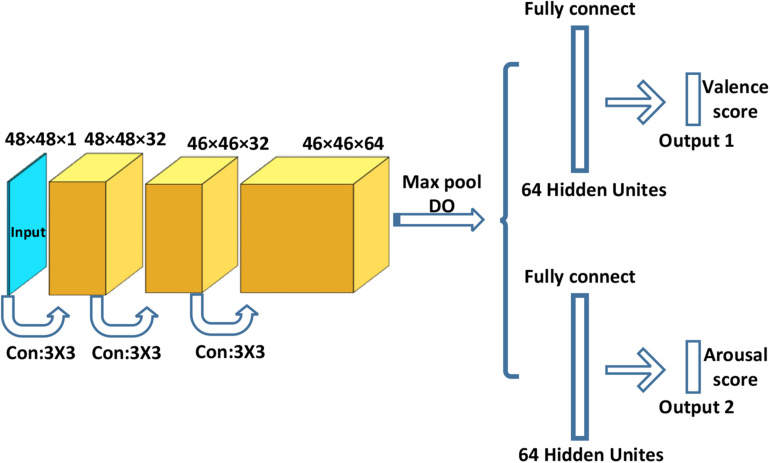
Multitask convolutional neural network. “DO” represents the dropout layer.

The size of the input images was 48 × 48 × 1 (grayscale images). A dropout layer with a deactivation rate of 0.5 was applied between the output and the dense layer to partially mitigate overfitting. The first, second, and third layers were convolution layers, and the fourth layer was a fully connected layer. The first layer has 32 convolution kernels with a size of 3 × 3 × 1. We used padding for the first convolutional layer. Padding is the addition of null pixels to increase the size of an image. Null pixels here refer to pixels with a value of 0. We used Keras to implement padding and CNN. Here, we have a 48 × 48 × 1 image and a 3 × 3 × 1 filter. With padding, the size of the first input image could be enlarged to 50 × 50 × 1, and the output of the convolutional layer (the second layer) could be 48 × 48 × 1, which preserves the same size as the original input image. The second layer had 32 convolution kernels with a size of 3 × 3 × 32. The third layer had 64 convolution kernels with a size of 3 × 3 × 32. The fourth layer was fully connected to 64 neurons. The final output layer had output valence and arousal scores for given emotion states. All convolutional layers and fully connected layers included a rectified linear unit (***ReLU***) as the activation function. Finally, the multitask CNN included two fully connected layers for separating valence and arousal scores. Figure 6 shows the architecture of the multitask CNN.

#### Emotion Computing Based on Facial Expression

The first branch of the fully connected layer was used to calculate the valence scores, and the second branch was used to calculate the arousal scores. The output scores were sent to a ***sigmoid*** function to minimize the cross-entropy loss. Equation (1) represents the loss function *L_n_*.

(1) $$L_{n}=-\sum_{i=1}^{m}\left(1-y_{ni}\log{\hat{y}}_{ni}\right)\log\left(1-{\hat{y}}_{ni}\right)$$

In equation (1), ***n*** represents the branch of the fully connected layer (when ***n*** is 1, *L*_*n*_ is the loss function of the valence branch; and when ***n*** is 2, *L*_*n*_ is the loss function of the arousal branch), *y*_*ni*_ represents the ground-truth labels for the *i^th^* sample, ${\hat{y}}_{ni}$ represents the ***sigmoid*** outputs for the *i^th^* sample, and ***m*** represents the size of the training sample.

Finally, we minimized the linear combination of *L*_1_ and *L*_2_. Equation (2) represents the linear combination of the loss functions.

(2) $$L=\sum_{i=1}^{2}\alpha_{i}L_{i}$$

In equation (2), α_*i*_ represents linear weights. Notably, if any of α_*i*_ is set to 0, the model returns to a single-task CNN model.

After emotion regression calculations, we classified emotions based on equation (3).

(3) $$r_{face}=\{{}_{lowS_{face}<0.5}^{highS_{face}\geq 0.5}$$

In equation (3), *r*_*face*_ represents the classification results of the valence or arousal dimension associated with a facial expression, and *S_face_* represents the scores of regression calculations. Thus, the valence and arousal dimension scores are dichotomized at high and low levels, and there are a total of four emotion categories: high valence and high arousal, high valence and low arousal, low valence and high arousal, low valence and low arousal.

### Electroencephalogram-Based Emotion Recognition

This subsection describes the emotion recognition methods in MindLink-Eumpy based on EEG signals. Here, we introduce the workflow of EEG-based emotion recognition. First, we used the Emotiv EPOC+ headset to record online EEG signals. Then, the multitaper method with fast Fourier transform (FFT) (Thomson, 1982) was used to extract the PSD features of the EEG signals. Finally, all the features were input into the SVM or LSTM for emotion recognition.

#### Electroencephalogram Data Processing and Feature Extraction

Previous studies (Alsolamy and Fattouh, 2016; Guzel Aydin et al., 2016; Jiang et al., 2019) have demonstrated that the PSDs of theta, alpha, beta, and gamma in the frontal, temporal, and occipital regions of the brain are highly related to human emotion. In this study, the features of EEG were extracted and selected based on these findings. In order to reduce the occurrence of artifacts, we first issued proper instructions to the subjects and repeatedly instructed the subjects to avoid blinking or moving their bodies during the experiment. Then, the filtering and multitaper (Thomson, 1982) techniques were used to remove the artifacts and keep the related neurological phenomenon intact in data processing.

It is worth noting that, in this study, we proposed two different EEG processing approaches for the online analysis and the offline analysis. Specifically, in the online analysis, we first remove artifacts through the function provided by the software development kit (SDK) of the Emotiv EPOC+ headset. Then, we used FFT to calculate PSDs, which is also provided by this equipment. It should be stressed that our EEG recording equipment Emotiv EPOC+ headset is designed for emotion recognition, and it can capture the EEG data from the following 14 channels located in the frontal, temporal, and occipital lobes: AF3, F3, F7, FC5, T7, P7, O1, AF4, F4, F8, FC6, T8, P8, and O2. However, in the offline analysis, the EEG data were bandpass filtered on five frequency bands (theta, slow alpha, alpha, beta, and gamma) from 4 to 45 Hz by finite impulse response (FIR) filters, and then the corresponding PSDs were obtained by FFT (overlap: 50%, time window: 1 s). In order to improve the accuracy in offline analysis of public databases, we selected 14 channels different from the online analysis: Fp1, T7, CP1, Oz, Fp2, F8, FC6, FC2, Cz, C4, T8, CP6, CP2, PO4. Finally, the PSDs of 14 channels and three symmetric pairs of channels (“T7–T8,” “Fp1–Fp2,” and “CP1–CP2”) were used as the EEG features.

#### Subject-Dependent Method Based on Support Vector Machine

After reading EEG signals from the above electrodes, five PSD features were extracted: theta (4 Hz < f < 8 Hz), slow alpha (8 Hz < f < 10 Hz), alpha (10 Hz < f < 12 Hz), beta (12 Hz < f < 30 Hz), and gamma (30 Hz < f < 45 Hz) features. The total number of EEG features was 70 (14 × 5 = 70). MindLink-Eumpy uses SVM-RFE (recursive feature elimination) to select optimal features by iteratively calculating feature weights of the linear SVM classifier and subsequently removing the 10% features with the lowest weights. Then, we split the selected features with 10-fold inner cross-validation for the training set (Duan et al., 2005). Following the facial expression modality tasks, we classified emotion according to equation (4).

(4) $$r_{EEG}=\{{}_{lowS_{EEG}<0.5}^{highS_{EEG}\geq 0.5}$$

In equation (4), *r*_*EEG*_ represents the classification result for the valence or arousal dimension in the EEG modality. *S*_*EEG*_ represents the scores of the regression calculations in the EEG modality.

#### Subject-Independent Method Based on Long Short-Term Memory

In this paper, we proposed an EEG-based subject-independent emotion recognition method based on LSTM. MindLink-Eumpy provides a well-trained model for this method. To implement this method, we constructed the features of the time sequence and performed regression calculations based on the LSTM. Since we applied supervised learning techniques to train the model, the data of the subjects in the experiment are all labeled and have the same distribution, resulting in the high accuracy of the experimental results. However, most of the subject-independent methods applied semi-supervised learning techniques or transfer learning techniques (Rodrigues et al., 2019; Li et al., 2020). This means that due to the domain differences between different subjects, the data in the model evaluation process cannot be labeled and its data distribution is different from the data in the model training process, which leads to a degradation in performance.

Here, we used the wavelet transform algorithm described above to extract features. Additionally, we regarded data every 10 s as a set of samples and sampled the data with an overlap rate of 50%. In the offline experiments using the MAHNOB-HCI database, 85 features were sampled per second. We picked 14 channels from the MAHNOB-HCI database and three symmetrical channel pairs of EEG data. For each channel, five PSD features are extracted from raw EEG data. Thus, the number of EEG features is (14 + 3) × 5 = 85. Each 10-s sample was used to construct a matrix with the size of 10 × 85, of which the first dimension is 10 and the second is 85. In this way, we avoided temporarily saving features in a one-dimensional vector unsuitable for the LSTM model.

The LSTM consisted of two LSTM layers, a fully connected layer and an output layer. The first LSTM layer contained 10 LSTM cells, each with 128 neurons. The second LSTM layer also contained 10 LSTM cells, but each cell had 64 neurons. The fully connected layer had 54 neurons, and the final output layer had two neurons that output the valence and arousal scores. Each of the abovementioned layers used a dropout rate of 0.5, which adopted the ***ReLU*** activation function and required data normalization. The mean square error was used as the loss metric for this LSTM network.

### Fusion Methods

By recording EEG signals and facial images through hardware devices (Emotiv EPOC+ headset and optical computer camera), MindLink-Eumpy toolbox reads and saves multimodal data and combines the predictions of the SVM (EEG modality) and CNN (facial expression modality) in decision level to improve the accuracy of emotion recognition. This subsection describes two decision-level fusion methods: the weight enumerator and AdaBoost method.

#### Weight Enumerator

We designed an enumerator to traverse weights in steps of 0.01 and find the optimal weights for the linear combination of two sub-classifiers. Equation (5) defines the linear combination.

(5) $$S_{enum}=\sigma S_{face}+\left(1-\sigma \right)S_{EEG}$$

In equation (5), σ ranges from 0 to 1, which represents the importance degree of the facial expression classifier; *S*_*face*_ and *S_EEG_* represent the prediction scores of the facial expression classifier and EEG-based classifier, respectively. The value of σ that achieves the highest accuracy is selected as the optimal weight for linear combination. Equation (6) defines the combined emotion classification relations.

(6) $$r_{enum}=\{{}_{lowS_{{}_{enum}}<0.5}^{highS_{{}_{enum}}\geq 0.5}$$

MindLink-Eumpy separately applies this fusion method in both the valence and arousal dimensions to classify emotion into four states.

#### AdaBoost

The second fusion method we used is the AdaBoost technique, which is to obtain the best parameters of ω_*j*_(*j*=1,2,….,*n*) for sub-classifiers. Equations (7) and (8) show the core mathematical formulas of AdaBoost.

(7) $$S_{boost}=\frac{1}{\left(1+\exp\left(-\sum_{j=1}^{n}w_{j}s_{j}\right)\right)}$$

(8) $$r_{boost}=\{{}_{lowS_{boost}<0.5}^{highS_{{}_{boost}}\geq 0.5}$$

In equation (7) and the below equations (9), (10), (11), (12), and (13), n represents the number of sub-classifiers, *s*_*j*_ ∈ {−1,1}_(*j*=1,2,…,*n*_ designates the outputs of the *j^th^* sub-classifier for the *i^th^* sample, and *S_boost_* represents the scores of fused emotion regression, which are calculated by the AdaBoost algorithm. For example, in this study, *s*_*1*_ represents an EEG-based sub-classifier and *s_2_* represents a facial expression sub-classifier. In equation (8), *r*_*boost*_ represents the fused emotion classification result.

The main process of AdaBoost is as follows. First, the training weights are initialized, as shown in equation (9):

(9) $$\alpha_{i}=\frac{1}{m}$$

α_*i*_ in equation (9) represents the weight of the *i^th^* sample, and ***m*** in equations (9) and (13) represents the size of the training sample. Each time AdaBoost updates sub-classifiers during model training, the sample data should be multiplied by the weights updated in the previous sub-classifier step. Equation (10) shows the mathematical formula for the error rate ε_*j*_.

(10) $$\varepsilon_{j}=\sum_{i=1}^{M}t_{i}\alpha_{i}$$

In equation (10), *t*_*i*_ is calculated from equation (11), and *y_i_* in equation (11) denotes the *i^th^* ground-truth label.

(11) $$t_{i}=\{{}_{1}^{0}{\;}_{s{\left(x_{i}\right)}_{j}\neq y_{i}}^{s{\left(x_{i}\right)}_{j}=y_{i}}$$

Then, we calculate the weights of the sub-classifier using equation (12).

(12) $$w_{j}=\frac{1}{2}\ln\left(\frac{1-\varepsilon_{j}}{\varepsilon_{j}}\right)$$

Next, we update weights for the next sub-classifier based on equation (13),

(13) $$\alpha_{j+1,i}=\{{}_{\frac{\alpha_{j,i}\exp\left(w_{j}\right)}{\sum_{i=1}^{m}\alpha_{j,i}\exp\left(w_{j}\right)}\;s{\left(x_{i}\right)}_{j}\neq y_{i}}^{\frac{\alpha_{j,i}\exp\left(-w_{j}\right)}{\sum_{i=1}^{m}\alpha_{j,i}\exp\left(-w_{j}\right)}s{\left(x_{i}\right)}_{j}=y_{i}}$$

where ***j*** represents the *j^th^* sub-classifier, α represents the weight of the *i^th^* sample for the *j^th^* sub-classifier, and *s*(*x*)_*j*_ represents outputs of the *j^th^* sub-classifiers for the *i^th^* sample.

MindLink-Eumpy also separately applies this fusion method in both the valence and arousal dimensions to classify emotion into two states (low or high state in each dimension).

## Experiments and Results

This section describes the experiments performed to evaluate MindLink-Eumpy. Three experiments were conducted in this study, including two offline experiments and one online experiment. In the offline experiments, for each database, subjects were selected according to the falling criteria: **(i)** the subject’s data contain both EEG and facial images; **(ii)** the subject’s ground-truth labels contain two states, including low and high in both valence and arousal dimensions.

### Offline Analysis

#### Experiments for the Subject-Dependent Methods

**FIGURE 7:**
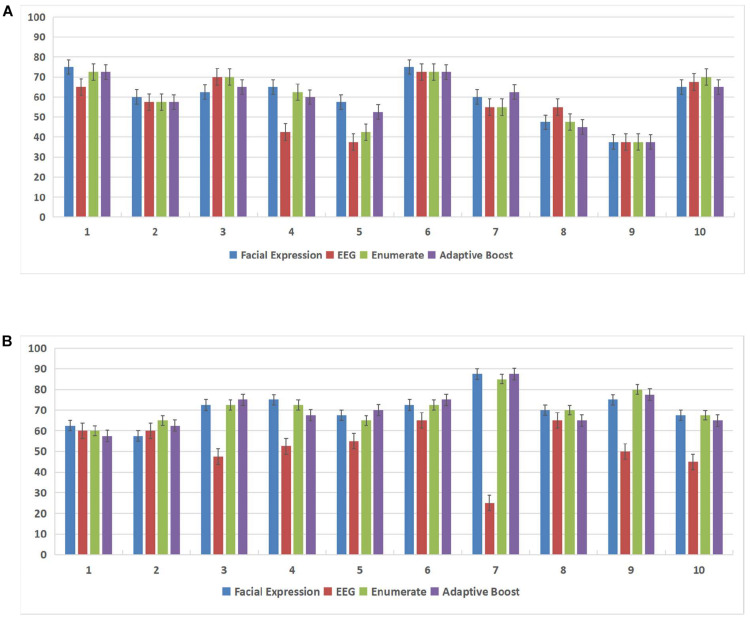
Accuracy for each subject in both the arousal and valence dimensions in the Database for Emotion Analysis Using Physiological Signals (DEAP) database. The X-axis of each subfigure represents the subject ID, and the Y-axis represents the accuracy (%). **(A)** Accuracy (%) for each subject in the arousal dimension in the DEAP database. **(B)** Accuracy (%) for each subject in the valence dimension in the DEAP database.

**FIGURE 8:**
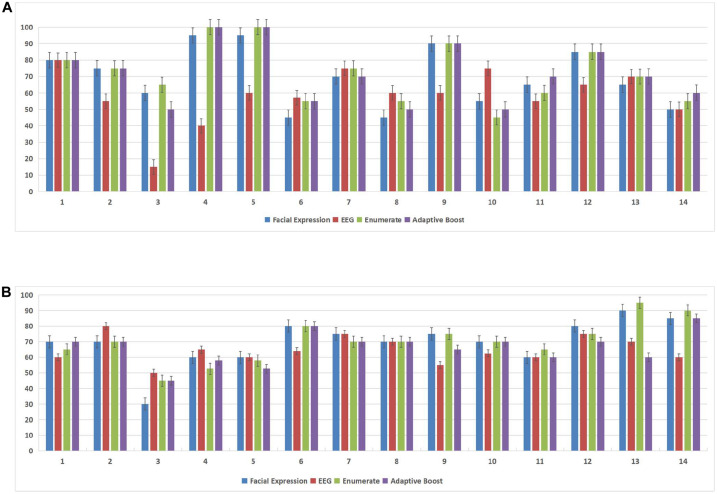
Accuracy for each subject in both the arousal and valence dimensions in the MAHNOB-HCI database. The X-axis of each subfigure represents the subject ID, and the Y-axis represents the accuracy (%). **(A)** Accuracy (%) for each subject in the arousal dimension in the MAHNOB-HCI database. **(B)** Accuracy (%) for each subject in the valence dimension in the MAHNOB-HCI database.

In this experiment, we used the DEAP database and the MAHNOB-HCI database to demonstrate the effectiveness of the subject-dependent methods based on multimodal emotion recognition. We chose 10 subjects in the DEAP database and 14 subjects in the MAHNOB-HCI database, then for each database, we randomly selected 20 trials of data of each subject as the training datasets, and the remaining 20 trials were used as the test datasets. Figure 7 and Table 2 show the offline experimental results (average values and accuracy thresholds) for the subject-dependent models in the DEAP database; and Figure 8 and Table 3 show the results in the MAHNOB-HCI database. The experimental results show that the first fusion method, the weight enumerator, achieved the highest accuracy in both the valence (in the DEAP database and the MAHNOB-HCI database) and arousal dimensions (in the MAHNOB-HCI database). The second fusion method, AdaBoost, also had a promising average accuracy, but the overall performance was lower than that of the weight enumerator, which was probably because of the less number of sub-classifiers (only has two modalities including facial expression and EEG signals). In addition, among the single-modal methods (that is, when there is only facial emotion recognition or EEG-based emotion recognition), the SVM in the valence dimension and CNN in the arousal dimension displayed promising accuracy. However, single-modal emotion recognition method was still less stable than the multimodal method. In this experiment, we observed that most subjects used the multimodal method to obtain higher accuracy, especially subjects 4 and 5 in the valence dimension in the MAHNOB-HCI and subjects 13 and 14 in the arousal dimension in the MAHNOB-HCI.

**TABLE 2:** Average accuracy (%) of subject-dependent models based on the DEAP database.

Target	Facial expression	EEG	Enumerator fusion	AdaBoost fusion
Valence	70.75 ± 7.67	52.50 ± 11.29	71.00 ± 7.00	70.25 ± 8.25
Arousal	60.50 ± 10.83	56.00 ± 12.46	58.75 ± 12.26	59.00 ± 10.74

*DEAP, Database for Emotion Analysis Using Physiological Signals; EEG, electroencephalogram.*

**TABLE 3:** Average accuracy (%) of subject-dependent models based on the MAHNOB-HCI database.

Target	Facial expression	EEG	Enumerator fusion	AdaBoost fusion
Valence	69.64 ± 14.07	64.75 ± 8.05	70.04 ± 12.81	66.11 ± 10.04
Arousal	69.64 ± 16.95	58.36 ± 15.86	72.14 ± 16.77	71.79 ± 16.97

*EEG, electroencephalogram.*

Furthermore, we conducted a normality test for these four methods (the SVM, CNN, weight enumerator, and AdaBoost methods). The data samples were considered normally distributed when the result was below 0.05; otherwise, we conducted another paired *t*-test procedure. During the *t*-test procedure, we considered that when the ***p*** value was lower than 0.05, the difference was statistically significant. The experiments were conducted based on the DEAP database and MAHNOB-HCI database. For the DEAP database, in the valence dimension, there was not only a significant difference (***p*** < 0.01) between the enumerator-based fusion results (weight enumerator) and the EEG-based results but also a significant difference (***p*** = 0.016) between the AdaBoost fusion results and the EEG-based results, but no significant difference was observed in the arousal dimension. For the MAHNOB-HCI database, no significant difference was observed in the valence dimension; but in the arousal dimension, there was a significant difference (***p*** = 0.045) between the AdaBoost fusion results and the EEG-based results.

#### Experiments for the Subject-Independent Methods

In the experiment for subject-independent methods, we used the EEG dataset collected from 30 subjects in the MAHNOB-HCI database to train and evaluate the LSTM model. We conducted the experiment by the following steps: Data from subject 1 to subject 20 were selected as the training set. After model training, we conducted an evaluation test using all data from subject 21 to subject 23. The experimental results show that the well-trained LSTM model achieved an accuracy of 78.56% and a recall rate of 68.18% in the valence dimension. Meanwhile, it achieved an accuracy of 77.22% and a recall rate of 69.28% in the arousal dimension. All the experimental results are shown in Table 4, including the training losses (Loss), validation loss, accuracy, recall rate, and root mean square error (RMSE). Table 4 shows that values of training losses for the valence and arousal dimensions were 3.16 and 2.17, respectively, and the validation losses were 3.35 and 3.30, respectively. Furthermore, the recall rates for the valence and arousal dimensions were 68.18% and 69.28%, respectively, and the RMSEs were 1.83 and 1.82, respectively.

**TABLE 4:** Experimental results of the subject-independent model on MAHNOB-HCI.

Dimension	Loss	Validation loss	Accuracy	Recall rate	RMSE
Valence	3.16	3.35	78.56%	68.18%	1.83
Arousal	2.17	3.30	77.22%	69.28%	1.82

*RMSE, Root mean square error.*

### Online Experiment

In the online experiment, we used the Emotiv EPOC+ headset and optical computer camera to record EEG data and facial images. Fifteen healthy subjects participated in the experiment, including eight males and seven females. The ages of the subjects ranged from 17 to 21 years old (mean = 20.27, SD = 1.24). Before the experiment, 40 videos for emotion elicitation were selected from YouTube. We manually divided these videos into two groups for calibration and evaluation experiments. The video clips ranged in duration from 70.52 to 195.12 s (mean = 143.04, SD = 33.50). During the experiments, we calibrated the position of the headset and the camera and ensured that the subjects were in a comfortable environment. Then, subjects were instructed to watch emotion-eliciting video clips and stay focused, remain calm, and avoid blinking or moving during the viewing process. After the end of each experimental trail, the subjects reported their emotion status in the valence and arousal dimensions through a questionnaire.

**FIGURE 9:**
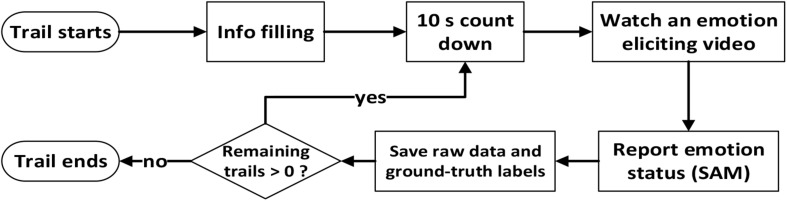
Workflow for one subject in the online experiment.

In the calibration process, we conducted experiments for data collection and ground-truth label calibration. We performed 20 trials for each subject. For the convenience of extracting data from specific subjects, each subject was required to provide personal information before the start of the first experimental trial so that MindLink-Eumpy toolbox could associate the information of each subject with the corresponding physiological data and ground-truth labels, such as name, age, and gender. At the beginning of each trial, a 10-s countdown appeared in the center of the computer screen to attract the subject’s attention. After the countdown, a video was presented in the screen to elicit the subject’s emotion. MindLink-Eumpy recorded four facial images and 10 groups of EEG signals per second and then saved the data in the database. At the end of each trial, each subject was required to assign SAM scale values for the valence and arousal scores. After clicking the “submit” button, the next trial started, and a 10-s countdown appeared again between the adjacent trials. Figure 9 presents the workflow of one trial for data collection in the online experiment.

In the evaluation experiments, a similar experimental trial process was used to evaluate the models. We used different videos to elicit the subject’s emotion. In each trial, we used four methods (the SVM, CNN, weight enumerator, and AdaBoost methods) to detect emotion. We calculated accuracy by comparing the predicted emotions and ground-truth labels.

**FIGURE 10:**
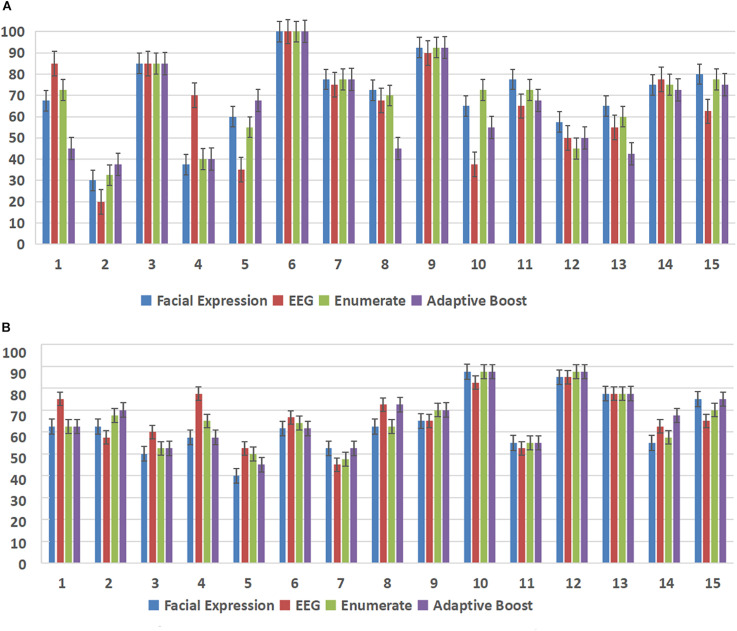
Accuracy (%) of each subject in both the arousal and valence dimensions in the online experiment. The X-axis of each subfigure represents the subject ID, and the Y-axis represents the accuracy (%). **(A)** Accuracy (%) of each subject in the arousal dimension in the online experiment. **(B)** Accuracy (%) of each subject in the valence dimension in the online experiment.

Figure 10 and Table 5 show the online experimental results (average values and thresholds of accuracy) for models of subject dependence. Notably, the multimodal methods achieved higher accuracy than the single-modal methods, except that the accuracy of the EEG-based SVM in the arousal dimension was higher than that of the enumerated fusion method.

**TABLE 5:** Average (%) accuracy of the subject-dependent models for online databases.

Target	Facial expression	EEG	Enumerate fusion	AdaBoost fusion
Valence	63.27 ± 12.68	66.44 ± 11.39	65.12 ± 11.78	66.27 ± 12.23
Arousal	69.50 ± 17.96	65.00 ± 21.53	68.50 ± 18.28	63.50 ± 19.47

*EEG, electroencephalogram.*

We also conducted a paired *t*-test for the online experiments. According to the experimental results, significant differences were observed both in valence and arousal dimensions between the AdaBoost fusion results and the facial expression results, where ***p*** = 0.014 in the valence dimension and ***p*** = 0.049 in the arousal dimension.

## Discussion and Conclusion

### Summary

This paper proposes MindLink-Eumpy, which is an open-source Python toolbox for multimodal emotion recognition. MindLink-Eumpy includes a series of tools for data collection, multimodal data processing, machine learning methods, and deep learning methods. Our aim of developing MindLink-Eumpy is to provide an extensible software framework for research and application in the field of emotion recognition.

First, MindLink-Eumpy implements an event-related paradigm that uses videos to elicit subject’s emotion. To record facial images and EEG information for subjects, MindLink-Eumpy implements tools to invoke computer cameras and headsets. Most importantly, MindLink-Eumpy implements a series of methods to overcome traditional difficulties in the field of emotion recognition: (i) two fusion methods are applied to combine SVM (EEG modality) and CNN (facial images) results to improve the accuracy of emotion recognition; (ii) a multitask CNN (facial images) based on transfer learning is used to overcome the overfitting phenomenon caused by lacking of image data; and (iii) LSTM based on EEG is used to implement a subject-independent emotion recognition technique. Finally, all the above methods have been tested and demonstrated to be effective. In particular, in experiments for the subject-independent methods, although the performance deteriorates when training data and validation data were completely heterogeneous, an acceptable accuracy was still maintained. Thus, this method has promising stability. Although MindLink-Eumpy is still in its infancy, it has the potential to become a benchmark toolbox in industrial and lab applications in the emotion recognition area.

### Analysis of the Advantages of MindLink-Eumpy

Advantages of MindLink-Eumpy mainly consist of three points: **(i)** a promising accuracy and subject independence for emotion recognition, **(ii)** a better robustness and scalability for software, **(iii)** a framework for online experimental paradigm and data storage medium.

Specifically, in this study, we proposed two approaches including facial expression and EEG for multimodal emotion recognition, each of which corresponds to a sub-classifier. Facial expression recognition approach is more accurate, but users may camouflage expressions in the real usage scenario, so an EEG emotion recognition approach is in use to fill the gap between the error associated with facial expressions and the Bayesian error in ground-truth emotion labels. To fuse multimodal information, we proposed two methods, weight enumerator and Adaboost, to improve the emotion recognition accuracy. Furthermore, we proposed another approach for subject-independent emotion recognition (based on LSTM in EEG modality) to make it suitable for more users. The subject-independent approach is independent of SVM and CNN approaches. Meanwhile, although feature-level fusion is more accurate theoretically, it is difficult to fuse spatial features (such as facial images) with temporal features (such as EEG information). Therefore, we applied decision-level fusion methods to ensure a better robustness mentioned above, which means that MindLink-Eumpy is able to keep running steadily even if there occurs errors in one equipment (such as camera, brain–computer interfaces, or other devices to be added). It is easier to add different modality information for multimodal emotion recognition using the methods of decision-level fusion in MindLink-Eumpy. Furthermore, MindLink-Eumpy provides a framework for online experiments. During the online experiments, MindLink-Eumpy stores physiological data into a folder system for future scientific research.

Although the performance was improved by information fusion, the superiority of the multimodal fusion over the single-modal approach did not show strong statistical significance in our results (e.g., when an independent two-sample *t*-test was performed on the accuracy distribution, the *p* values in Tables 2, 3 are not always less than 0.05). In many emotion experiments [e.g., Alsolamy and Fattouh (2016)], it can be found that high volatility is associated with facial expressions because subjects can trick the machine by imitating certain facial expressions. For this problem, the gap between the error related to facial expressions and the Bayesian errors of true emotion detection generally can be filled by adding information sources (e.g., EEG) (Li et al., 2019). For the experiments on DEAP and MAHNOB-HCI databases, the subjects were asked to behave normally rather than mimic certain facial expressions, which may be the main reason that we could not find strong statistical evidence indicating significant improvement after fusion. Furthermore, there are only 10 subjects in the DEAP and 14 subjects in the MAHNOB-HCI that meet our experimental requirements. The limited sample size may be another reason why the results are not statistically significant.

### Comparison With Other Methods in the Literature

The main functions of MindLink-Eumpy comprise data collection and storage, data preprocessing, feature extraction and visualization, and emotion recognition. This toolbox is integrated with our methods for multimodal emotion recognition. Moreover, it is able to facilitate scientific research on multimodal emotion recognition. Compared with other studies, MindLink-Eumpy complements the existing research in some ways.

From the perspective of multimodal emotion recognition methods, it is important to achieve a promising accuracy and make models subject independent. MindLink-Eumpy combines facial expression and EEG for a promising accuracy and provides an LSTM model based on EEG for subject independence. Li et al. (2019) combined EEG and facial expression data to optimize emotion recognition algorithms. Three types of movie clips (positive, neutral, and negative) were utilized for emotion data collection, and LSTM was utilized for decision-level fusion and capturing temporal dynamics of emotion, which yield a concordance correlation coefficient (CCC) of 0.625 ± 0.029.

Under the circumstance that we divide emotions into two classifications in both valence and arousal dimension (four categories in total), MindLink-Eumpy is demonstrated to be more accurate and subject independent in emotion recognition. In a binary classification of valence and arousal dimensions, our experimental results demonstrated that MindLink-Eumpy has a promising performance in both offline analysis and real-time detection. For instance of experiments on MAHNOB-HCI database, the average accuracy of 68.50% was achieved in arousal dimension in the study of Koelstra and Patras (2013), while in our study, it reached 72.14%. Taking the DEAP database as an example, the study of He et al. (2017) achieved an average accuracy of 70.90% in valence dimension, while the average of accuracy in our study reached 72.14%. Chen et al. (2017) combined EEG information, peripheral physiological signals, and facial video to obtain promising accuracy (77.57% for four emotion classifications). This study obtained higher accuracy and subject independence than the work of Buitelaar et al. (1999). However, it lacked facial expression modality and needed wearable equipment to acquire all of the physiological signals, which means that it is stringent to apply their method in practice.

Furthermore, from the perspective of data and software, not only research on emotion recognition needs more physiological data storage medium, feature extractors, and experimental framework, but practical application scenario needs functions of data streaming and real-time visualization. MindLink-Eumpy provides a folder system for data storage and integrates a series of tools for preprocessing and feature extraction of facial images and EEG signals. Besides, MindLink-Eumpy is suitable for devices including brain–computer interfaces and cameras for real-time data acquisition and visualization. Buitelaar et al. (2018) proposed a toolbox named MixedEmotion that provided audio processing, text processing, and video processing for multimodal emotion analysis. MixedEmotion is well developed and practical application oriented, but it lacks physiological information for intuitive feedback. It mainly focuses on enterprise application but does not contribute to scientific research on human neuroscience. Soleymani et al. (2017) proposed a toolbox named TEAP for the signal processing of EEG, GSR, EMG, skin temperature, respiration pattern, and blood volume pulse information, which expanded the application scope of multimodal emotion analysis. The authors of Soleymani et al. (2017) had tried to replicate some methods of other articles and demonstrate the effectiveness of feature extraction function of TEAP. But they had not proposed their original methods for emotion recognition. Generally speaking, MindLink-Eumpy provides a framework for scientific research and application with our original approaches for subject-independent emotion recognition. Compared with other works, MindLink-Eumpy promotes research in the area of emotion recognition.

Compared with a subject-dependent approach, LSTM-based achieved higher accuracies. Here are two reasons for this situation. First, the inherent difference between SVM and LSTM may be one of the reasons for the performance difference between them. A previous study (Nath et al., 2020) compared the different performances of LSTM in subject-dependent and subject-independent experiments. Their results in subject-independent recognition showed that the average accuracy rates of valence and arousal dimensions were 70.31% and 69.53%, respectively. The experimental results are basically consistent with our conclusion that the LSTM-based method achieved an accuracy of 78.56% in the valence dimension and an accuracy of 77.22% in the arousal dimension for the subject-independent recognition. Second, the outliers of data also hinder the high performance of subject-dependent methods. In this study, although we paid much attention to data preprocessing, we still found some strange but not dirty data different from normal ones. For example, the PSD values of some trails of certain subjects remain low. It is hard to filter all these outliers of data for SVM. Therefore, in the case of the subject dependence, a well-trained SVM model may pay too much attention to outliers, thereby reducing the imitation effect of the model. For the case of the subject independence, abundant data enable the LSTM model to focus on the universality of features of all subjects rather than outliers, thereby eliminating the effects caused by relatively few outliers. In the future, we will try to analyze the abnormal data and remove outliers.

### Potential Applications

MindLink-Eumpy could provide a potential software benchmark for emotion recognition in industry applications. Thus, there are various potential applications based on the technologies and frameworks of MindLink-Eumpy. In the medical field, emotion recognition plays an important role in the treatment of children with autism (Buitelaar et al., 1999), hearing-impaired children (Gu et al., 2019), patients with depression (Punkanen et al., 2011), etc., In the field of intelligent driving, research has focused on the behaviors of drivers affected by emotions (Roidl et al., 2014). Extreme emotions might lead to improper operations or even traffic accidents, thereby endangering drivers and passengers. Emotion recognition technologies can also be applied for supervisory care, including baby care, intensive care, Alzheimer’s care, etc., Overall, MindLink-Eumpy has promising application prospects.

### Limitations

As a software toolbox, MindLink-Eumpy has limitations. First, MindLink-Eumpy only provides tools for EEG information and facial images. Other widely used physiological data, such as eye movement signals and electrocardiograph (ECG) information, are not currently compatible with MindLink-Eumpy. In addition, although multimodal emotion recognition methods outperform single-modal methods and the average accuracy of multimodal methods is high, the results of our experiments did not display strong statistical significance. In the experiments, as described by the Hawthorne effect, subjects may tend to display biased facial expressions due to their awareness of being observed. Moreover, it is challenging to record a large EEG dataset because of the volatility of conductive media in brain-computer interface (BCI) and the lack of subjects.

### Future Work

In the future, we will attempt to add new data modalities to improve multimodal emotion recognition and implement new tools suitable for different hardware devices. In single-modal EEG-based emotion recognition, we will implement semi-supervised machine learning algorithms for cross-subject detection. Furthermore, we will try to use eye movement signal to measure domain differences among subjects and implement methods for the feature-level fusion of eye movement signals and EEG information.

## Data Availability Statement

The raw data supporting the conclusions of this article will be made available by the authors, without undue reservation.

## Ethics Statement

The studies involving human participants were reviewed and approved by the Ethics Committee of South China Normal University. The patients/participants provided their written informed consent to participate in this study. Written informed consent was obtained from the individual(s) for the publication of any potentially identifiable images or data included in this article.

## Author Contributions

JP: conceptualization, supervision, project administration, and funding acquisition. JP, RL, and YL: methodology and validation. RL, ZC, and BW: software. RL, WH, and ZC: formal analysis. JP, LQ, and YL: investigation. RL and XL: writing – original draft preparation. JP, YL, YY, and LQ: writing – review and editing. RL, WH, and BW: visualization. All authors contributed to the article and approved the submitted version.

## Conflict of Interest

The authors declare that the research was conducted in the absence of any commercial or financial relationships that could be construed as a potential conflict of interest.
